# Comparison of lingual oscillometric and invasive blood pressure measurement in dogs under general anaesthesia

**DOI:** 10.1111/jsap.70163

**Published:** 2026-07-12

**Authors:** C. Kyle, J. Deutsch

**Affiliations:** ^1^ Langford Vets University of Bristol Langford Bristol BS40 5DU UK

## Abstract

**Objectives:**

The objective of the study was to evaluate agreement between tongue‐based oscillometric and invasive arterial blood pressure in anaesthetised dogs, assess performance against American College of Veterinary Internal Medicine and Association for the Advancement of Medical Instrumentation/European Society of Hypertension/International Organization for Standardization validation criteria and examine proportional bias overall and by body size and phenotype.

**Materials and Methods:**

Prospective observational study of 89 client‐owned dogs undergoing general anaesthesia. Simultaneous tongue‐based oscillometric and invasive arterial systolic, mean and diastolic pressures were recorded at 5‐minute intervals (1780 paired observations). Agreement was assessed using Bland–Altman analysis and performance judged against American College of Veterinary Internal Medicine and Association for the Advancement of Medical Instrumentation/European Society of Hypertension/International Organization for Standardization criteria. Proportional bias (difference vs. mean pressure) was evaluated with linear mixed‐effects models with dogs as a random effect, with body size (≤10 kg *vs.* >10 kg) and phenotype (chondrodystrophic *vs.* non‐chondrodystrophic) as interaction terms.

**Results:**

American College of Veterinary Internal Medicine criteria were met for mean and diastolic but not systolic pressure; no measure met Association for the Advancement of Medical Instrumentation/European Society of Hypertension/International Organization for Standardization thresholds. Limits of agreement for mean and diastolic pressures were wide. Systolic pressure showed magnitude‐dependent underestimation, whereas mean and diastolic pressures did not. Underestimation increased with systolic pressure in dogs >10 kg and in non‐chondrodystrophic dogs, whereas no clear pressure‐dependent trend was seen in dogs ≤10 kg or in chondrodystrophic dogs. Transient tongue swelling occurred in one dog; no other tongue‐related adverse events were recorded.

**Clinical Significance:**

Tongue‐based oscillometry achieved acceptable agreement for mean and diastolic arterial pressures according to American College of Veterinary Internal Medicine criteria but did not meet Association for the Advancement of Medical Instrumentation/European Society of Hypertension/International Organization for Standardization validation standards. However, limits of agreement remained wide, and tongue‐based oscillometric blood pressure should be interpreted cautiously and in conjunction with clinical context, rather than as a direct substitute for invasive blood pressure measurements. When invasive monitoring is unavailable, it remains useful for following trends in mean and diastolic pressure. Systolic values should be interpreted cautiously, particularly at higher pressures in dogs >10 kg or non‐chondrodystrophic dogs.

## INTRODUCTION

Arterial blood pressure measurement represents an important monitoring tool during anaesthesia. Anaesthetic drugs, continuous mechanical ventilation and surgical stimulation can all alter vascular tone and cardiac output, predisposing to hypo‐ or hypertension which can impair organ perfusion and subsequently function (Almeida et al., 2014).

Maintaining mean arterial blood pressure (MAP) above commonly accepted minimum targets (≥60 to 70 mmHg in small animals) is used as a surrogate for adequate organ perfusion, particularly for the brain, kidney and spinal cord (Ruffato et al., 2015). Current monitoring guidelines recommend continuous invasive blood pressure (IBP) monitoring, or regular non‐invasive blood pressure (NIBP) monitoring if IBP is not feasible (Grubb et al., 2020). Such monitoring supports early recognition and treatment of haemodynamic compromise (Skelding & Valverde, 2020).

In small animals, IBP monitoring requiring placement of an arterial catheter is considered the gold standard (MacFarlane et al., 2010). However, it is technically demanding, requires specialised equipment and carries risks including infection, haematoma, haemorrhage and vascular injury (Scheer et al., 2002). These limitations restrict the widespread use of IBP, particularly in general practice.

Indirect NIBP monitoring, involving a cuff with an inflatable bladder placed over a peripheral artery on the limb or tail of the dog, is widely used as an alternative. Compared to Doppler‐based measurements, oscillometry appears to provide better agreement with IBP and meets more of the validation criteria recommended by the American College of Veterinary Internal Medicine (ACVIM) (Vachon et al., 2014). Placement of NIBP cuffs on the limb or tail can, however, be problematic in patients with conformational abnormalities such as chondrodystrophic breeds or when surgical positioning limits access to these sites.

The tongue, with its rich vascular supply and accessibility during anaesthesia, has been proposed as an alternative site for oscillometric BP monitoring. In research Beagles, good agreement between tongue‐based oscillometry and IBP measurements for MAP and diastolic arterial pressure (DAP) was demonstrated, though correlation with systolic arterial pressure (SAP) was less consistent (Kim et al., 2022). Similar results were confirmed in client‐owned anaesthetised dogs, with MAP and DAP closely estimated in animals >5 kg (Kim et al., 2023).

These data suggest that the tongue may represent a reliable alternative site for NIBP monitoring in anaesthetised dogs. However, further evaluation in a larger clinical cohort, including assessment of proportional bias, detection of hypotension and potential effects of body size and phenotype, is warranted.

This study aimed to evaluate the accuracy of tongue‐based oscillometric BP (NIBPt) against IBP in client‐owned anaesthetised dogs. The primary objective was agreement for SAP, MAP and DAP measured against ACVIM validation criteria (Brown et al., 2007) and device validation benchmarks developed for human blood pressure monitors by the Association for the Advancement of Medical Instrumentation (AAMI)/European Society of Hypertension (ESH)/International Organization for Standardization (ISO) collaboration (Stergiou et al., 2018). Secondary objectives examined proportional bias and potential effect modification by body size and phenotype.

## MATERIALS AND METHODS

### Animals

The study was conducted at Langford Vets Small Animal Referral Hospital at the University of Bristol, between July 2023 and January 2025. Ethical approval for this study was obtained from the Animal Welfare and Ethical Review Body (VIN‐23‐016).

Any dog undergoing a surgical procedure could be included in the study after written and informed owner consent was obtained for oscillometric cuff placement on the tongue. The decision to place an arterial catheter was made at the discretion of the supervising board‐certified veterinary anaesthetist depending on the severity of the patient's condition and procedure. For dogs undergoing hemilaminectomy where arterial catheter placement is not routinely performed at the author's institution, additional informed owner consent was obtained.

Dogs were excluded if surgical procedures precluded access to the head (*e.g*. hemimandibulectomy), if arterial catheterisation was unsuccessful, or if marked anaesthetic instability occurred (*e.g*. impending cardiac arrest). No exclusions were applied based on signalment, American Society of Anaesthesiologists (ASA) physical status classification or anaesthetic protocol. All dogs were categorised according to ASA classification following pre‐anaesthetic examination, and anaesthetic management was supervised by a European specialist in Veterinary Anaesthesia and Analgesia.

### IBP monitoring

Following induction of anaesthesia, an arterial catheter (Jelco® IV catheter, Smiths Medical ASD, Inc., Minneapolis, MN, USA) was aseptically placed in the dorsal pedal, auricular or coccygeal artery after standard skin preparation. Catheters were secured with tape to minimise movement artefact during monitoring, and the insertion site was recorded.

In theatre, the arterial catheter was connected *via* non‐compliant tubing to a fluid‐filled pressure transducer system. The transducer was zeroed to atmospheric pressure prior to connection and positioned at the level of the right atrium to provide a hydrostatic reference point. In lateral recumbency, this was achieved by aligning the transducer with the sternum at the approximate location of the heart base; in dorsal recumbency, alignment was with the point of the shoulder.

Arterial waveform quality was assessed using a square‐wave (fast‐flush) test and classified as normal, overdamped or underdamped according to the response of the trace. IBP (SAP, MAP, DAP) was continuously displayed on a multiparameter monitor along with oxygen saturation (SpO_2_), respiratory rate, end‐expiratory carbon dioxide and inhalant concentration, oesophageal temperature, heart rate (HR) and rhythm.

### Experimental protocol

Tongue‐based oscillometric BP monitoring was performed following the technique described (Kim et al., 2022, 2023). With the tongue in a neutral position, its circumference rostral to the lingual frenulum was measured to guide cuff selection (Fig 1).

**FIG 1:**
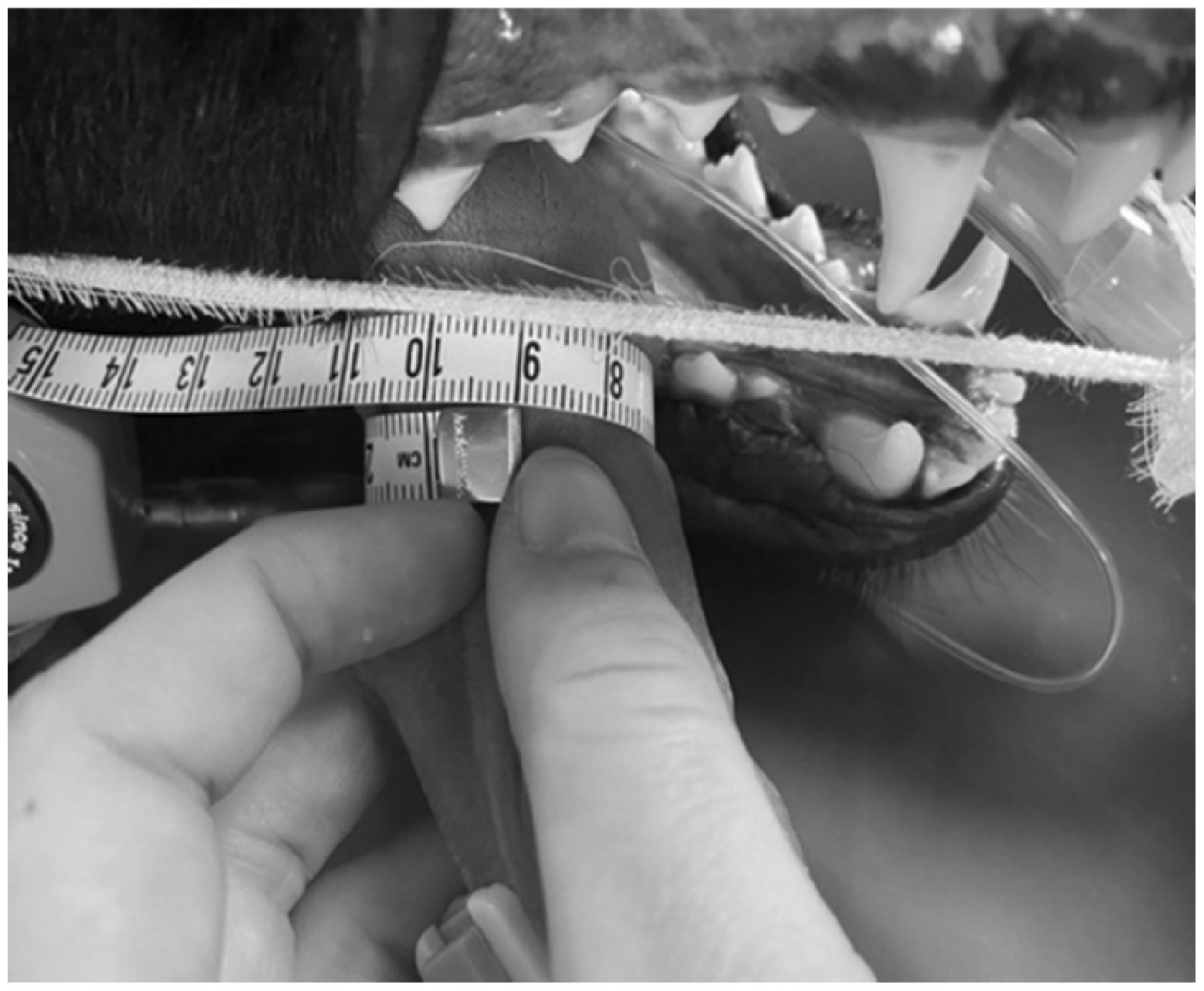
Measurement of the tongue circumference. The tape measure is placed rostral to the lingual frenulum and the circumference is measured with the tongue in a neutral position.

Disposable, single‐lumen (Soft Disposable, SunTech, NC USA) cuffs were selected based on the measured tongue circumference using a predefined, non‐overlapping range in which cuff bladder width approximated 40% of the tongue circumference (Table 1). The ranges were established after measurement of the bladder width of each cuff size. Proper fit was confirmed by ensuring that the index arrow on the cuff aligned within the manufacturer's reference lines. The cuff was placed rostral to the lingual frenulum and fitted snugly, avoiding folding or visible compression of the tongue. The tongue was then allowed to rest in a neutral position during measurement. Cuffs were connected to an oscillometric BP monitor (SunTech Vet20, NC USA) which was programmed to obtain measurements automatically every 5 minutes.

**Table 1:** Tongue circumference ranges and corresponding tongue cuff sizes for non‐invasive blood pressure monitoring

Tongue circumference range (mm)	Cuff size
<55	1
55 to 73	2
74 to 94	3
95 to 114	4
>114	5

Tongue circumference was measured rostral to the lingual frenulum with the tongue in a neutral position (Figure 1). Cuff size ranges were defined after measuring cuff bladder width to approximate 40% of tongue circumference for each cuff size and then assigning non‐overlapping circumference ranges to each cuff.

### Data recording

NIBPt, IBP and HR were recorded every 5 minutes throughout anaesthesia. Arterial waveform quality (normal, overdamped or underdamped) was documented at initial connection of the arterial line and every 30 minutes thereafter. Any corrective interventions to the arterial line (*e.g*. flushing, disconnection, repositioning) were noted.

Subject signalment (sex, bodyweight, breed), intraoperative drug administration (agent, dose, time) and any adverse events were recorded. In the event of NIBPt measurement failure, the cuff was repositioned and the measurement repeated. Monitoring continued until the end of surgery, at which point the cuff was removed prior to recovery; removal of the arterial catheter was at the discretion of the attending anaesthetist.

Data points were excluded if: (i) NIBPt failed to generate a reading despite cuff repositioning or repeat measurement; (ii) NIBPt‐derived HR did not match the arterial pulse rate from the IBP waveform; (iii) noticeable artefact was present on the IBP waveform; (iv) cardiovascular instability was evident (*e.g*. arrhythmia, pulse deficits, sudden haemodynamic collapse); or (v) IBP and NIBPt values were not obtained within the same 5‐minute recording interval.

### Statistical analysis

Statistical analysis was performed using Microsoft Excel (version 16.78.3; Microsoft Corporation, Redmond, WA, USA), JASP (version 0.95.1, Intel) and RStudio (Posit Software, PBC, Boston, MA). In line with AAMI/ESH/ISO guidance for blood pressure measuring device validation, the target sample size was set at ≥85 dogs (Stergiou et al., 2018).

Agreement between IBP and NIBPt was assessed for SAP, MAP and DAP using Bland–Altman analysis, with IBP treated as the reference standard. Bias was defined as IBP − NIBPt (mmHg), such that positive values indicate NIBPt underestimation of IBP and negative values indicate overestimation. For each pressure, pair‐level mean bias, precision (standard deviation (SD) of the paired differences) and 95% limits of agreement (LoA = bias ±1.96 × SD) were calculated with 95% confidence intervals (CIs) derived using standard *t*‐based methods. Because multiple paired measurements were obtained per dog, pair‐level Bland–Altman summaries were interpreted descriptively and did not adjust for within‐dog clustering. Formal inference on proportional bias and subgroup effects was based on mixed‐effects models including dog as a random intercept.

Bland–Altman plots (difference *vs*. mean) were constructed for all pressures. Proportional bias was assessed using linear mixed‐effects models regressing the paired difference (IBP − NIBPt) on centred mean pressure, with dog as a random intercept. Random slopes for mean pressure were evaluated and retained only if they improved model fit. Model assumptions were evaluated using residual‐*versus*‐fitted and *Q*–*Q* plots.

Acceptable performance of the NIBPt was judged against ACVIM criteria (minimum eight animals; ≥50% of paired measurements within ±10 mmHg and ≥80% within ±20 mmHg of IBP) (Brown et al., 2007) and AAMI/ESH/ISO criteria (human benchmark; ≥85 subjects and ≥85% of paired measurements within ±10 mmHg of IBP) (Stergiou et al., 2018).

As an additional clinically orientated descriptive assessment, pair‐level NIBPt MAP performance for detecting IBP‐defined hypotension was assessed at MAP thresholds of <60, <65 and <70 mmHg. Sensitivity, specificity, positive predictive value (PPV) and negative predictive value (NPV) were calculated using IBP MAP as the reference.

To explore potential effect modification, dogs were stratified by body size (≤10 kg *vs*. >10 kg) and by phenotype (chondrodystrophic *vs*. non‐chondrodystrophic). Body weight was assessed because weight has previously been suggested to affect NIBPt performance. These analyses were interpreted as hypothesis‐generating. Within each subgroup, pair‐level Bland–Altman summaries (bias, precision (SD), LoA and proportions within ±10 and ±20 mmHg) were calculated. Interaction terms (mean × weight group; mean × phenotype) were added to the mixed‐effects models and evaluated by likelihood ratio tests (LRTs).

As a descriptive sensitivity analysis, subject‐level Bland–Altman summaries were generated using per‐dog means; no regression was undertaken at the subject level.

## RESULTS

### Study population and data yield

A total of 120 dogs were enrolled. Arterial catheter placement failed in 28 cases; in two dogs, the wrong cuff size was selected; and in one dog, insufficient data were recorded, resulting in 89 dogs for analysis contributing to 1780 paired observations. Baseline demographic, procedural and monitoring characteristics are summarised in Table 2. Agreement and proportional bias results are reported overall and by prespecified strata, with validation thresholds (Table 3) and a subject‐level sensitivity (Table S1).

**Table 2:** Demographic, procedural and monitoring characteristics of 89 anaesthetised client‐owned dogs undergoing invasive and tongue‐based oscillometric blood pressure monitoring.

Variable	Value
Sex/reproductive status, *n* (%)	
Male neutered (MN)	29 (33%)
Female neutered (FN)	27 (30%)
Male entire (ME)	19 (21%)
Female entire (FE)	14 (16%)
Age (months)	67 ± 30 (range 15 to 157)
Body weight (kg)	13.9 ± 14.6 (range 4.0 to 98.4)
Body weight category, *n* (%)	
≤10 kg	45 (51%)
>10 kg	44 (49%)
Breed, *n* (%)	
Dachshund	34 (38%)
Other chondrodystrophic	2 (2%)
Other non‐chondrodystrophic	53 (60%)
Procedure, *n* (%)	
Cardiology intervention	1 (1%)
Hemilaminectomy	58 (65%)
Other neurological	10 (11%)
Soft tissue	20 (23%)
Duration of data collection (minutes)	102 ± 48 (range 25 to 215)
Arterial catheter site, *n* (%)	
Dorsal pedal artery	56 (63%)
Auricular artery	31 (35%)
Coccygeal artery	1 (1%)
Not recorded	1 (1%)
Arterial waveform quality, *n* (%)	
Normal	38 (43%)
Abnormal (over‐/under‐damped or mixed)	42 (47%)
Not assessed	9 (10%)
Tongue circumference (mm)	85 ± 23 (range 53 to 174)
NIBPt cuff size, *n* (%)	
Size 1	1 (1%)
Size 2	35 (39%)
Size 3	27 (30%)
Size 4	17 (19%)
Size 5	9 (10%)

Values are presented as *n* (%) for categorical variables and mean ± standard deviation (SD) with range for continuous variables. Percentages may not sum to 100% due to rounding. Chondrodystrophic status was classified as Dachshund, other chondrodystrophic breeds or non‐chondrodystrophic breeds based on skeletal phenotype.

**Table 3:** Pair‐level (non‐clustered) Bland–Altman agreement between invasive blood pressure (IBP) and tongue‐based oscillometric blood pressure (NIBPt), stratified by body size and phenotype.

Stratum	Dogs (*N*)	Pairs (*n*)	Measure	Bias^#^ (mmHg)	Precision^‡^ (mean bias SD)	LoA low	LoA high	% NIBP within ±10 mmHg of IBP^§^	% NIBP within ±20 mmHg of IBP^§^	Criteria meeting ACVIM^¶^	Criteria meeting AAMI/ESH/ISO^††^
All dogs	89	1780	SAP	4.8	21.7	−37.7	47.4	40	71.1	No	No
			MAP	3.2	12.4	−21.1	27.4	69	90.6	Yes	No
			DAP	−0.8	13.9	−28.1	26.5	68	89.9	Yes	No
Weight ≤10 kg	45	790	SAP	−2.5	21.9	−45.5	40.4	41	75.9	No	No
			MAP	1.3	14.3	−26.7	29.4	67	88.1	Yes	No
			DAP	−2.5	16.0	−34.0	28.9	67	87.5	Yes	No
Weight >10 kg	44	990	SAP	10.7	19.7	−27.9	49.2	39	67.3	No	No
			MAP	4.6	10.4	−15.7	24.9	71	92.5	Yes	No
			DAP	0.5	11.8	−22.6	23.7	69	91.8	Yes	No
Chondrodystrophic	36	648	SAP	−2.6	19.6	−40.9	35.8	45	79.6	No	No
			MAP	2.4	11.5	−20.2	25.0	74	93.5	Yes	No
			DAP	−1.5	12.5	−25.9	22.9	69	92.1	Yes	No
Non‐chondrodystrophic	53	1132	SAP	9.0	21.8	−33.6	51.7	38	66.3	No	No
			MAP	3.6	12.8	−21.5	28.8	67	88.9	Yes	No
			DAP	−0.5	14.7	−29.2	28.3	68	88.6	Yes	No

Bias is defined as (IBP − NIBPt), precision (SD of paired differences), 95% limits of agreement (LoA) and the proportions of paired measurements within ±10 and ±20 mmHg of IBP are shown for SAP, MAP and DAP across all dogs and within prespecified strata (≤10 kg *vs*. >10 kg; chondrodystrophic vs. non‐chondrodystrophic). Columns indicate the number of dogs contributing pairs, total pairs analysed and whether ACVIM or AAMI/ESH/ISO criteria were met.

^#^ Bias = IBP − NIBPt (mmHg); positive values indicate NIBPt underestimation of IBP

^‡^ Precision (SD) = standard deviation of paired differences; LoA = bias ± 1.96 × SD

^§^ “% within ±10/±20 mmHg” are pair‐level proportions relative to IBP

^¶^ ACVIM criterion: ≥50% within ±10 mmHg and ≥80% within ±20 mmHg (minimum eight animals)

^††^ AAMI/ESH/ISO shown as a human device validation benchmark (study target ≥85 subjects); numeric pass/fail is reported for transparency

### Agreement overview

Bland–Altman plots (Fig 2) illustrate paired differences (IBP − NIBPt) across the measurement range. SAP showed a positive bias, indicating underestimation by NIBPt, with wide LoA (bias +4.81 mmHg, LoA −37.73 to +47.35 mmHg), whereas MAP and DAP exhibited smaller biases with moderate dispersion (MAP bias +3.17 mmHg, LoA −21.10 to +27.43 mmHg; DAP bias −0.83 mmHg, LoA −28.12 to +26.46 mmHg). Because these Bland–Altman summaries are pair‐level and repeated measurements were obtained from each dog, they should be interpreted descriptively rather than as estimates based on independent observations. Within‐dog clustering was addressed in the mixed‐effects proportional bias analyses.

**FIG 2:**
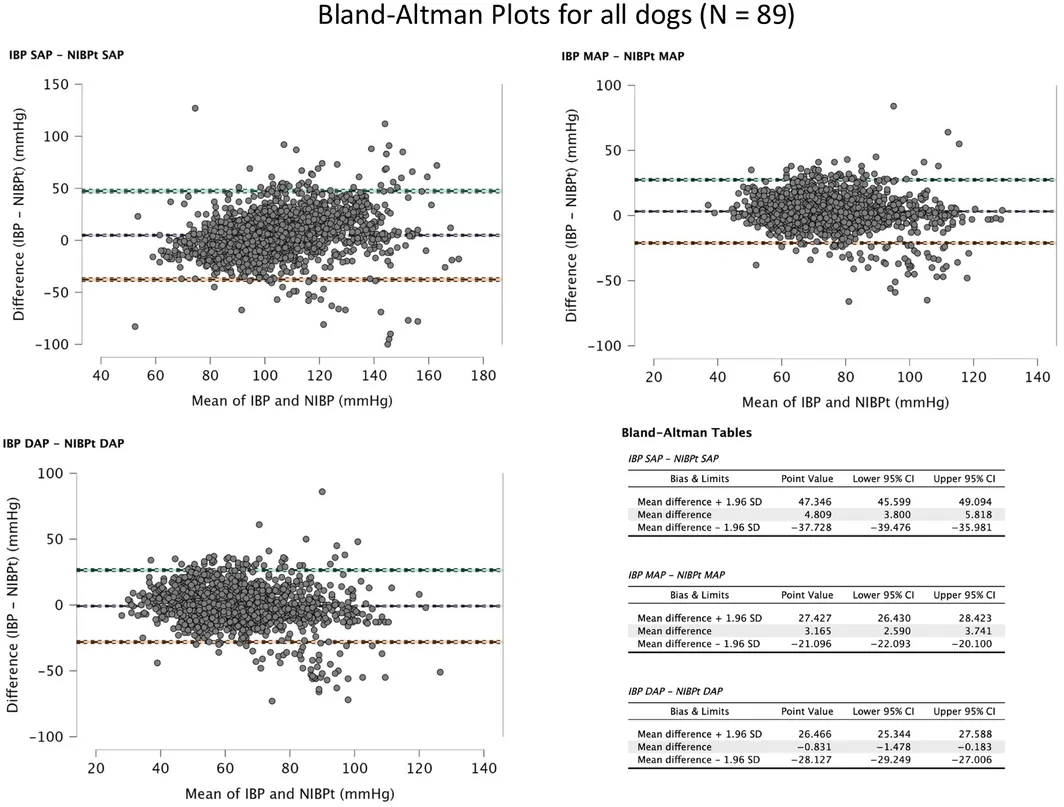
Bland–Altman plots comparing invasive arterial blood pressure (IBP) with tongue‐based oscillometric blood pressure (NIBPt) in all dogs for systolic (SAP), mean (MAP) and diastolic (DAP) pressures. Points represent paired measurements. The central dashed line is the mean difference (bias = IBP − NIBPt); outer dashed lines are the mean difference ± 1.96 standard deviations (SD) of the differences (95% limits of agreement, LoA). Thin flanking dashes indicate the 95% confidence intervals (CIs) for the bias and each LoA. Axes in mmHg. The Bland–Altman summary table lists the point estimate and 95% CI for the mean difference and for each LoA (presented as “mean difference ±1.96 SD”).

### Mixed‐effects proportional bias (all dogs, Supporting Information Model S1)

Mixed‐effects modelling indicated magnitude‐dependent error for SAP but not for MAP or DAP. In clinical terms, as systolic pressure increased, NIBPt tended to underestimate IBP by a greater amount. For each 1 mmHg increase in mean SAP, NIBPt read approximately 0.22 mmHg lower than IBP (SAP slope + 0.22; 95% CI 0.07 to 0.37). MAP and DAP slopes were not statistically different from zero (95% CIs included 0). Model diagnostics were acceptable, with greater dispersion at higher SAP and more uniform scatter for MAP/DAP.

### Detection of hypotension

Diagnostic performance of NIBPt MAP for detecting IBP‐defined hypotension is summarised in Table 4. Sensitivity increased as the MAP threshold rose, from 64.2% at <60 mmHg to 82.0% at <70 mmHg, while specificity decreased from 86.7% to 71.8%. Across thresholds, negative predictive value was high (88.8% to 96.0%), whereas positive predictive value was lower (32.5% to 59.3%).

**Table 4:** Hypotension was defined using invasive blood pressure mean arterial pressure (IBP MAP) thresholds of <60, <65 and <70 mmHg

IBP MAP threshold (mmHg)	TP	FP	FN	TN	Sensitivity (%)	Specificity (%)	PPV (%)	NPV (%)
< 60	104	216	58	1402	64.2	86.7	32.5	96.0
< 65	264	297	107	1112	71.2	78.9	47.1	91.2
< 70	487	334	107	852	82.0	71.8	59.3	88.8

Tongue‐based oscillometric mean arterial pressure (NIBPt MAP) was considered positive when below the same threshold. Values are descriptive pair‐level estimates; repeated measurements were obtained from individual dogs and should not be interpreted as independent subjects.

TP True positive, FP False positive, FN False negative, TN True negative, PPV Positive predictive value, NPV Negative predictive value.

### Subgroup agreement

Weight‐ and phenotype‐stratified Bland–Altman plots are presented (Figs S1 and S2). For MAP and DAP, dogs >10 kg had better precision (smaller SD of the differences) and narrower LoA than dogs ≤10 kg; chondrodystrophic dogs showed precision advantages over non‐chondrodystrophic dogs. SAP demonstrated wider LoA in all strata. Full details of bias, precision, LoA and proportions within ±10/±20 mmHg are summarised in Table 3. The precision and LoA patterns reflect dispersion only and do not imply differences in proportional bias.

### Mixed‐effects interaction: weight (Supporting Information Model S2)

For SAP, proportional bias differed by body size (LRT *P =* .001): in dogs >10 kg, NIBPt increasingly underestimated IBP as SAP rose (+0.44 per 1 mmHg, 95% CI 0.24 to 0.65), whereas dogs ≤10 kg showed no proportional bias (slope −0.02; 95% CI −0.22 to 0.18). There was no strong statistical evidence of weight interaction for MAP (*P =* .44) or DAP (*P =* .43).

### Mixed‐effects interaction: phenotype (Supporting Information Model S3)

Using phenotype as chondrodystrophic *versus* non‐chondrodystrophic, proportional bias differed for SAP (LRT *P =* .01): in non‐chondrodystrophic dogs, NIBPt increasingly underestimated IBP across the systolic range (+0.27 per 1 mmHg, 95% CI 0.07 to 0.46), whereas chondrodystrophic dogs showed no proportional bias (slope +0.12, 95% CI −0.12 to 0.36). There was no strong statistical evidence of phenotype interaction for MAP (*P =* .10) or DAP (*P =* .17).

### Validation against external criteria

Accuracy thresholds met for the overall dataset and for the subgroups (≤10 kg *vs*. >10 kg; chondrodystrophic *vs*. non‐chondrodystrophic) are summarised in the validation table (Table 3). ACVIM criteria were met for MAP and DAP in the overall analysis and across subgroups, whereas SAP did not meet ACVIM criteria in any stratum. The AAMI/ESH/ISO threshold was not met for any measure or subgroup.

### Subject‐level (per dog) analysis

Subject‐level Bland–Altman estimates based on per‐dog means (Table S1) were consistent with the pair‐level findings.

### Complications and adverse events

Complications included four haematomas (after unsuccessful catheter placement or following catheter removal) and one episode of transient tongue swelling/erythema after NIBPt measurement. Concurrent tongue placement of the pulse oximeter probe and NIBPt cuff caused transient loss of SpO_2_ readings during cuff inflation.

No other adverse events related to catheter or cuff placement were observed, and all dogs recovered uneventfully from anaesthesia.

## DISCUSSION

This study evaluated the agreement between NIBPt and IBP in 89 anaesthetised dogs. Validation of NIBP techniques in small animals is commonly assessed against criteria established by the ACVIM and by human device standards from the AAMI/ESH/ISO collaboration. The ACVIM recommends that differences between IBP and NIBP not exceed 10 mmHg in at least 50% of measurements and 20 mmHg in at least 80% (Brown et al., 2007). The AAMI/ESH/ISO sets stricter requirements, with devices considered acceptable if ≥85% of measurements fall within 10 mmHg, in a validation study sample size of 85 subjects (Stergiou et al., 2018). In the context of these benchmarks, ACVIM validation criteria were met for MAP and DAP but not for SAP; none of the pressure variables met the AAMI/ESH/ISO benchmark. These findings align with prior canine studies in which MAP and DAP outperform SAP (Kim et al., 2022, 2023). Compared with those samples (*n* = 8 and *n* = 45), the present dataset of 89 dogs improves the precision of estimates and meets the minimum sample size requirement in AAMI/ESH/ISO validation guidance.

In the whole cohort, ACVIM criteria were achieved for MAP and DAP, but the LoA were wide and only about two‐thirds of pairs fell within ±10 mmHg (Table 3). Consequently, meeting ACVIM criteria should not be interpreted as evidence that NIBPt is interchangeable with IBP for individual clinical decisions. One in three single readings may deviate by >10 mmHg, and thresholds for intervention (*e.g*. MAP 60 to 70 mmHg) should therefore be applied with care. The hypotension detection analysis supported this caution (Table 4): NIBPt MAP had high NPV across thresholds of <60 to <70 mmHg, but lower PPV, indicating that low NIBPt MAP readings may suggest hypotension that is not confirmed by IBP. Mixed‐effects models showed strong statistical evidence for proportional bias in SAP only and no overall proportional bias for MAP or DAP. Together, these findings support reliance on repeated measurements and trends rather than single values for MAP and DAP; SAP interpretation requires caution, particularly at higher pressures.

Body size has been proposed as an effect modifier of NIBPt accuracy, with prior work suggesting better performance of MAP and DAP in dogs >5 kg (Kim et al., 2023). As the present dataset had too few dogs to stratify at 5 kg (≤5 kg, *n* = 7) and an imbalanced remainder, a 10‐kg cut‐point was adopted (≤10 kg, *n* = 45; >10 kg, *n* = 44) to enable a more informative comparison while still probing whether size modifies agreement. Phenotype (chondrodystrophic *vs*. non‐chondrodystrophic) was also examined as the tongue may prove a more accessible site in chondrodystrophic breeds where conformational limb abnormalities or surgical positioning impair appropriate cuff placement and fit. Establishing whether agreement differs by phenotype therefore has direct practical relevance. The subgroup analysis should be interpreted cautiously, as the study was not designed to determine the mechanisms underlying body size or phenotype effects. Further studies specifically evaluating these subgroups and potential underlying mechanisms are needed.

In stratified analyses, precision for MAP and DAP (smaller SD of differences, narrower LoA) was better in dogs >10 kg and in chondrodystrophic dogs. However, this did not alter their proportional bias; MAP and DAP values were not systematically over‐ or underestimated relative to other groups. By contrast, mixed‐effects interaction models indicated that effect modification was selective to SAP. In larger dogs (>10 kg), SAP demonstrated statistically significant proportional bias compared to smaller dogs (≤10 kg). Non‐chondrodystrophic dogs showed a similar pattern of SAP underestimation by NIBPt, whereas chondrodystrophic dogs showed no proportional bias. Despite these subgroup patterns, SAP failed to meet ACVIM criteria in any stratum and no subgroup met AAMI/ESH/ISO accuracy. MAP and DAP met ACVIM thresholds only. Taken together, these findings support the use of NIBPt for monitoring trends in MAP and DAP, while SAP should be interpreted with caution.

Oscillometric SAP consistently shows poorer agreement with IBP, with inaccuracies persisting regardless of cuff site (Garofalo et al., 2012; Sawyer et al., 2004). In the present data, SAP failed to meet ACVIM criteria and demonstrated positive proportional bias. The mixed‐effects models showed that NIBPt underestimation increased progressively as systolic pressure rose, consistent with previous studies (McMurphy et al., 2006). These SAP‐specific patterns may reflect known properties of oscillometry: MAP is anchored at the point of maximal cuff oscillation, whereas SAP and DAP are algorithmically extrapolated (Walter et al., 2025). In addition, systolic error tends to worsen at higher pressures (Sawyer et al., 2004) and with increased vascular resistance or waveform damping, leading to systematic underestimation (MacFarlane et al., 2010). Further studies evaluating the effects of drugs on systemic vascular resistance and of arterial pressure‐waveform quality on NIBPt could better characterise the changes observed here.

Beyond dogs, cross‐species investigations support the tongue as a practical oscillometric site. In pigs, lingual MAP met ACVIM criteria, while SAP and DAP did not (Makepeace et al., 2024). In horses, tongue‐based measurements met ACVIM criteria across all pressures and outperformed thoracic limb placement, particularly during hypotension (Parra Martinez et al., 2024). Considered alongside the present canine data, this suggests that trend monitoring of MAP is the most robust parameter for NIBPt in settings without IBP.

This study has several limitations. Anaesthetic protocols were not standardised, which likely influenced peripheral vascular tone and oscillometric signal quality. Potential confounders such as varied drug administrations, surgical positioning, treatment of hypotension, heart rate, rhythm irregularity and arterial waveform characteristics were not analysed. As 65% of analysed dogs underwent hemilaminectomy, findings should be generalised cautiously to other procedure types and anaesthetic populations. However, this reflects real‐world anaesthesia practice, as does the exclusion of 28 dogs in which arterial catheter placement was unsuccessful. This practical limitation of IBP monitoring highlights the need for alternative monitoring sites when arterial access is not achievable.

Arterial waveform quality was optimal in only 43% of subjects. Suboptimal waveform quality may have contributed to reduced agreement for SAP and DAP; however, MAP remains accurate even with damping artefacts (Michard, 2024), supporting the validity of these MAP results. In addition, IBP was measured from clinically accessible arterial sites, rather than from the lingual artery itself. Lingual arterial catheterisation is not routinely performed and would not have been practical alongside tongue cuff placement. Consequently, some disagreement, particularly for SAP, may reflect physiological differences in pulse‐wave transmission or local vascular characteristics between arterial sites rather than oscillometric error alone.

Cuff fit also affects accuracy; narrow or loose cuffs tend to overestimate and wide or tight cuffs underestimate IBP (Skelding & Valverde, 2020). A width of 30% to 40% of appendage circumference is recommended (Valtonen & Eriksson, 1970). In this study, cuff width was standardised using tongue circumference‐based ranges and cuffs were positioned rostral to the lingual frenulum, in line with previous descriptions of tongue NIBP in dogs (Kim et al., 2022, 2023). However, intra‐anaesthetic movement and repositioning of the tongue or cuff may still have altered fit and contributed to variability in NIBPt readings. In one French Bulldog, tongue swelling was observed at the end of the procedure after NIBPt measurement; this was transient and self‐limiting without clinical adverse effect. The tongue should be checked regularly when using this technique, particularly in brachycephalic dogs, where lingual swelling could contribute to upper airway obstruction during recovery.

Analytical considerations: To maintain comparability within the existing NIBPt veterinary literature (Kim et al., 2022, 2023; Makepeace et al., 2024; Parra Martinez et al., 2024), Bland–Altman analyses were reported at the pair level using the “multiple measurements per individual” approach. These summaries were descriptive and did not adjust for within‐dog clustering, as the 1780 paired observations were derived from 89 dogs rather than independent subjects. The associated standard errors and confidence intervals should therefore be interpreted cautiously. Within‐dog clustering and proportional bias were instead addressed using mixed‐effects models which formed the main inferential analysis. A subject‐level Bland–Altman sensitivity (per‐dog means) yielded similar qualitative conclusions. Further work could model pressure‐dependent variance and regression‐based LoA for repeated‐measures designs, particularly when proportional bias is present.

Further work should evaluate tongue‐based oscillometry across specific surgeries and positions, compare tongue‐based monitoring with other NIBP sites and Doppler in larger clinical populations and explore algorithmic refinements to reduce proportional bias. Given the observed magnitude‐dependent error for SAP, regression‐based LoA or range‐specific performance summaries may be informative in future studies. Stratifying agreement by IBP waveform quality, assessing performance during bradycardia and rhythm irregularity and prospectively testing clinical decision rules (*e.g*. detection of IBP MAP <60 to 70 mmHg) would directly address bedside utility.

It is important to recognise the limitations of the ACVIM validation framework itself. ACVIM guidance was developed for conscious animals and hypertension screening rather than intra‐anaesthetic monitoring. Wide LoA are common, even when ACVIM criteria have been met, reducing the reliability of individual measurements in clinical practice (Rysnik et al., 2013). There are no validation guidelines specific to general anaesthesia for cuff NIBP in humans or animals; AAMI/ESH/ISO thresholds are useful as comparative benchmarks rather than setting‐specific standards.

Tongue‐based NIBP in anaesthetised dogs satisfied ACVIM validation criteria for MAP and DAP but not SAP and did not satisfy the stricter AAMI/ESH/ISO benchmark. However, the wide LoA indicate that NIBPt should be interpreted cautiously for clinical decisions. NIBPt may be suitable for monitoring trends in MAP and DAP when IBP is unavailable. NIBPt SAP should be interpreted with caution, particularly at higher pressures and in dogs >10 kg or non‐chondrodystrophic dogs where proportional bias is evident.

### Author contributions

**J. Deutsch:** Conceptualization; methodology; supervision; writing – review and editing. **C. Kyle:** Data curation; investigation; validation; formal analysis; writing – original draft; software.

### Conflict of interest

The authors declare no conflict of interests.

### Funding

This research received no external funding.

## AI disclosure statement

No AI generative tools were used in the writing of this manuscript.

## Supporting information


**Supporting Information S1.** Statistical analysis and diagnostics (mixed‐effects proportional bias).

**Fig S1A:** Bland–Altman plots comparing invasive arterial blood pressure (IBP) with tongue‐based oscillometric blood pressure (NIBPt) in dogs ≤10 kg for systolic arterial blood pressure (SAP), mean arterial blood pressure (MAP) and diastolic arterial blood pressure (DAP). Points represent paired measurements. The central dashed line is the mean difference (bias = IBP − NIBPt); outer dashed lines are the mean difference ± 1.96 standard deviations (SD) of the differences (95% limits of agreement, LoA). Thin flanking dashes indicate the 95% confidence intervals (CIs) for the bias and each LoA. Axes in mmHg. The Bland–Altman summary table lists the point estimate and 95% CI for the mean difference and for each LoA (presented as “mean difference ±1.96 SD”.
**Fig S1B:** Bland– Altman plots comparing invasive arterial blood pressure (IBP) with tongue‐based oscillometric blood pressure (NIBPt) in dogs >10 kg for systolic arterial blood pressure (SAP), mean arterial blood pressure (MAP) and diastolic arterial blood pressure (DAP). Points represent paired measurements. The central dashed line is the mean difference (bias = IBP − NIBPt); outer dashed lines are the mean difference ± 1.96 standard deviations (SD) of the differences (95% limits of agreement, LoA). Thin flanking dashes indicate the 95% confidence intervals (CIs) for the bias and each LoA. Axes in mmHg. The Bland–Altman summary table lists the point estimate and 95% CI for the mean difference and for each LoA (presented as “mean difference ±1.96 SD”).
**Fig S2A:** Bland–Altman plots comparing invasive arterial blood pressure (IBP) with tongue‐based oscillometric blood pressure (NIBPt) in non‐chondrodystrophic dogs for systolic arterial blood pressure (SAP), mean arterial blood pressure (MAP) and diastolic arterial blood pressure (DAP). Points represent paired measurements. The central dashed line is the mean difference (bias = IBP − NIBPt); outer dashed lines are the mean difference ± 1.96 standard deviations (SD) of the differences (95% limits of agreement, LoA). Thin flanking dashes indicate the 95% confidence intervals (CIs) for the bias and each LoA. Axes in mmHg. The Bland–Altman summary table lists the point estimate and 95% CI for the mean difference and for each LoA (presented as “mean difference ± 1.96 SD”).
**Fig S2B:** Bland–Altman plots comparing invasive arterial blood pressure (IBP) with tongue‐based oscillometric blood pressure (NIBPt) in chondrodystrophic dogs for systolic arterial blood pressure (SAP), mean arterial blood pressure (MAP) and diastolic arterial blood pressure (DAP). Points represent paired measurements. The central dashed line is the mean difference (bias = IBP − NIBPt); outer dashed lines are the mean difference ± 1.96 standard deviations (SD) of the differences (95% limits of agreement, LoA). Thin flanking dashes indicate the 95% confidence intervals (CIs) for the bias and each LoA. Axes in mmHg. The Bland–Altman summary table lists the point estimate and 95% CI for the mean difference and for each LoA (presented as “mean difference ± 1.96 SD”).

**Table S1:** Subject‐level (per‐dog) Bland–Altman agreement between invasive blood pressure (IBP) and tongue‐based oscillometric blood pressure (NIBPt): Bias is defined as IBP − NIBPt (mmHg). For each dog and pressure (SAP, MAP, DAP), the per‐dog mean difference was computed; bias, precision (SD), and 95% limits of agreement (LoA) were derived from these per‐dog means. “% of dogs ≤10/≤20 mmHg” denotes the proportion of dogs whose per‐dog mean absolute difference from IBP was within the stated threshold. ACVIM and AAMI/ESH/ISO columns are provided for context at the subject level; formal device‐level validation was performed on the pair‐level dataset (Table 3). Strata include all dogs and subgroups by body size (≤10 kg *vs*. >10 kg) and phenotype (chondrodystrophic *vs*. non‐chondrodystrophic).

## Data Availability

The data that support the findings of this study are available from the corresponding author upon reasonable request.
