# Supplementary material for: Comparison of lingual oscillometric and invasive blood pressure measurement in dogs under general anaesthesia
Source: J Small Anim Pract. 2026 Jul 12;67(11):1082–90. doi: 10.1111/jsap.70163 (PMC13728962; doi:10.1111/jsap.70163)
Supplement: Supplementary file 1 [file JSAP-67-1082-s002.pdf]

Bland-Altman Plots for Dogs ≤10 kg (N = 45)

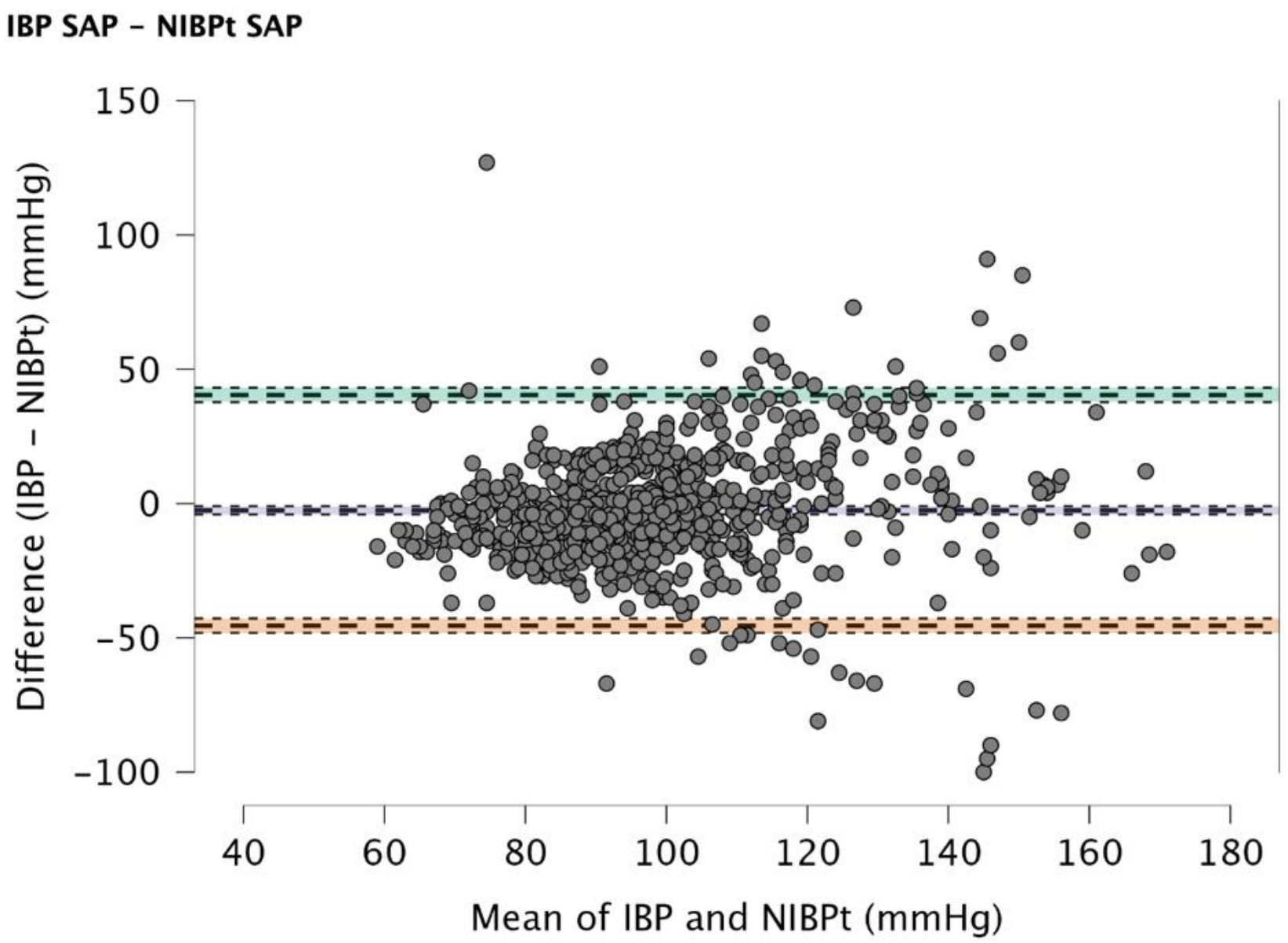

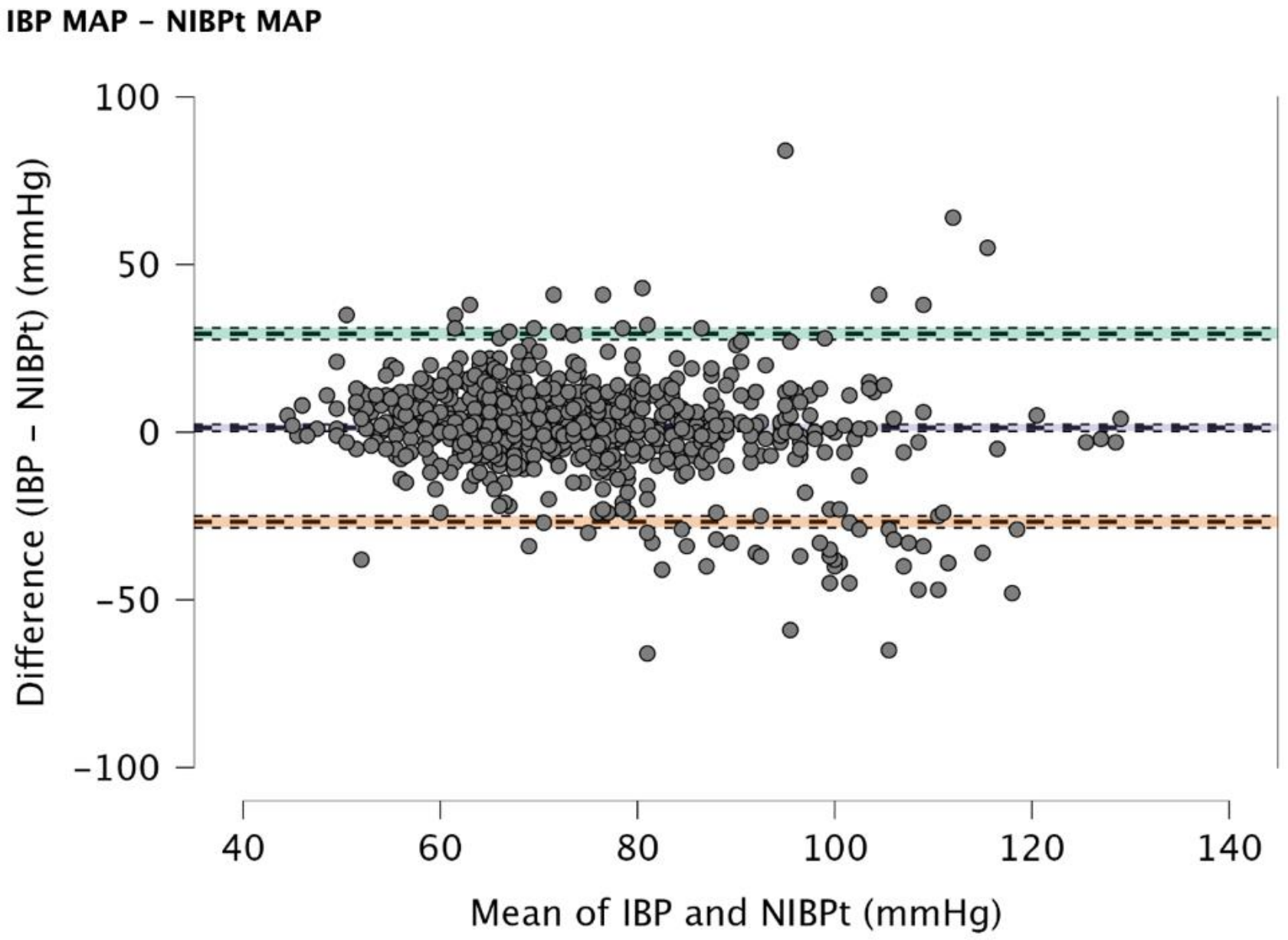

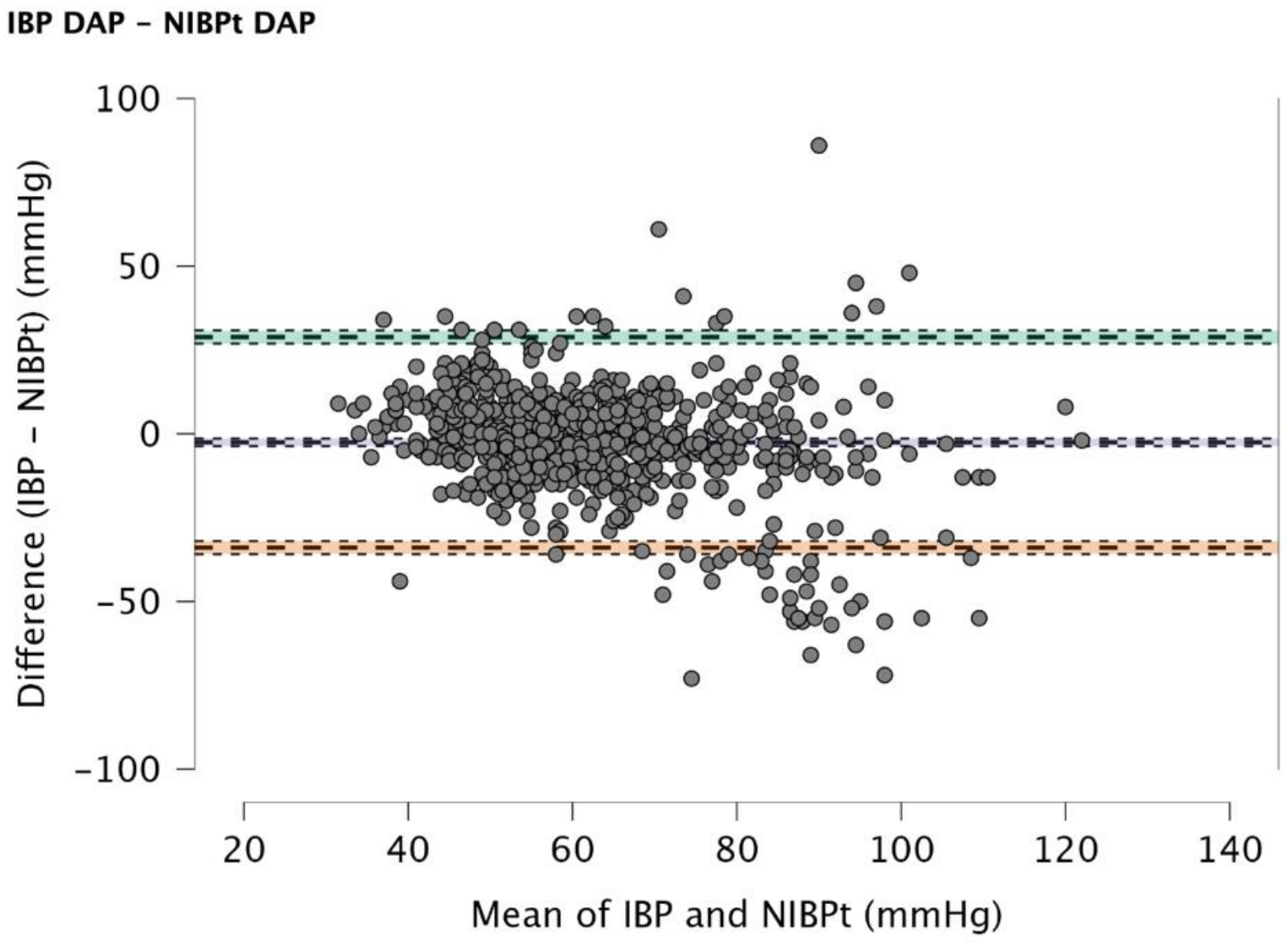

Bland-Altman Tables

IBP SAP – NIBPt SAP

Bias & Limits	Point Value	Lower 95% CI	Upper 95% CI
Mean difference + 1.96 SD	40.429	37.778	43.080
Mean difference	−2.528	−4.059	−0.997
Mean difference - 1.96 SD	−45.485	−48.136	−42.833

IBP MAP – NIBPt MAP

Bias & Limits	Point Value	Lower 95% CI	Upper 95% CI
Mean difference + 1.96 SD	29.385	27.653	31.116
Mean difference	1.332	0.332	2.331
Mean difference - 1.96 SD	−26.721	−28.453	−24.990

IBP DAP – NIBPt DAP

Bias & Limits	Point Value	Lower 95% CI	Upper 95% CI
Mean difference + 1.96 SD	28.865	26.925	30.805
Mean difference	−2.545	−3.665	−1.425
Mean difference - 1.96 SD	−33.955	−35.895	−32.015

Supplementary Figure 1A: ***Bland–Altman plots comparing invasive arterial blood pressure (IBP) with tongue-based oscillometric blood pressure (NIBPt) in dogs ≤10 kg for systolic arterial blood pressure (SAP), mean arterial blood pressure (MAP) and diastolic arterial blood pressure (DAP).*** *Points represent paired measurements. The central dashed line is the mean difference (bias = IBP − NIBPt); outer dashed lines are the mean difference ±1.96 standard deviations (SD) of the differences (95% limits of agreement, LoA). Thin flanking dashes indicate the 95% confidence intervals (CIs) for the bias and each LoA. Axes in mmHg. The Bland–Altman summary table lists the point estimate and 95% CI for the mean difference and for each LoA (presented as "mean difference ±1.96 SD").*

Bland-Altman Plots for Dogs >10 kg (N = 44)

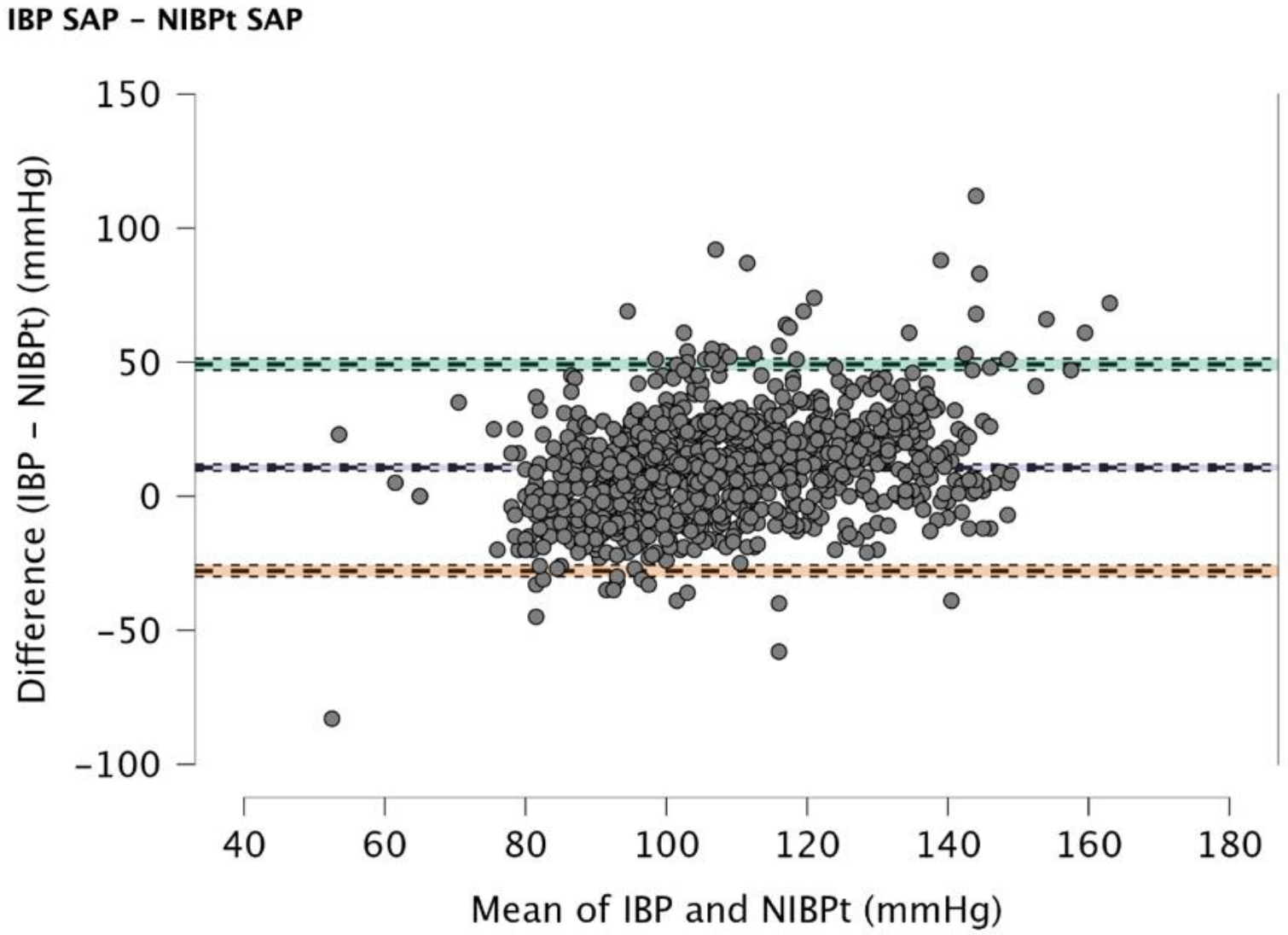

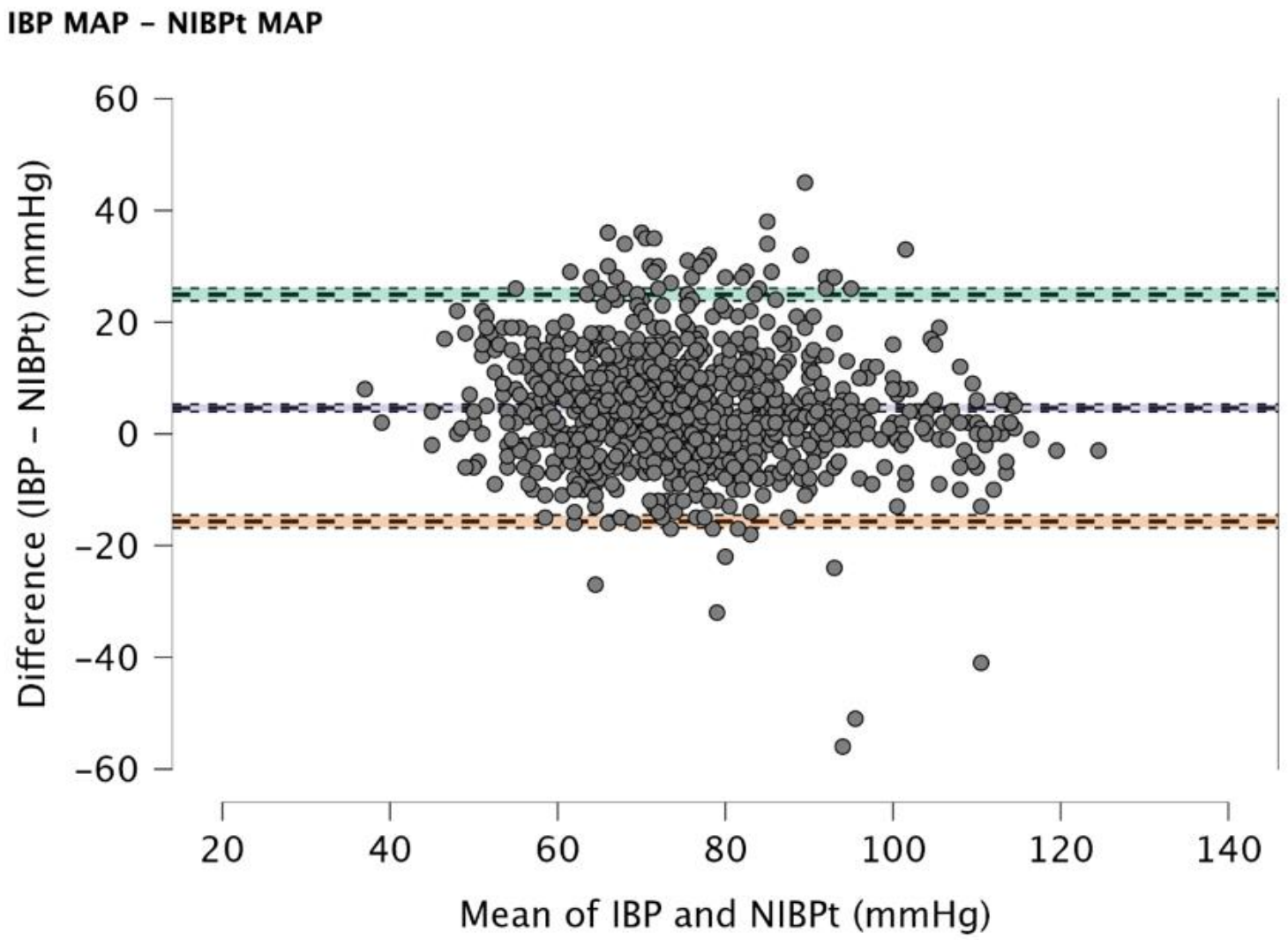

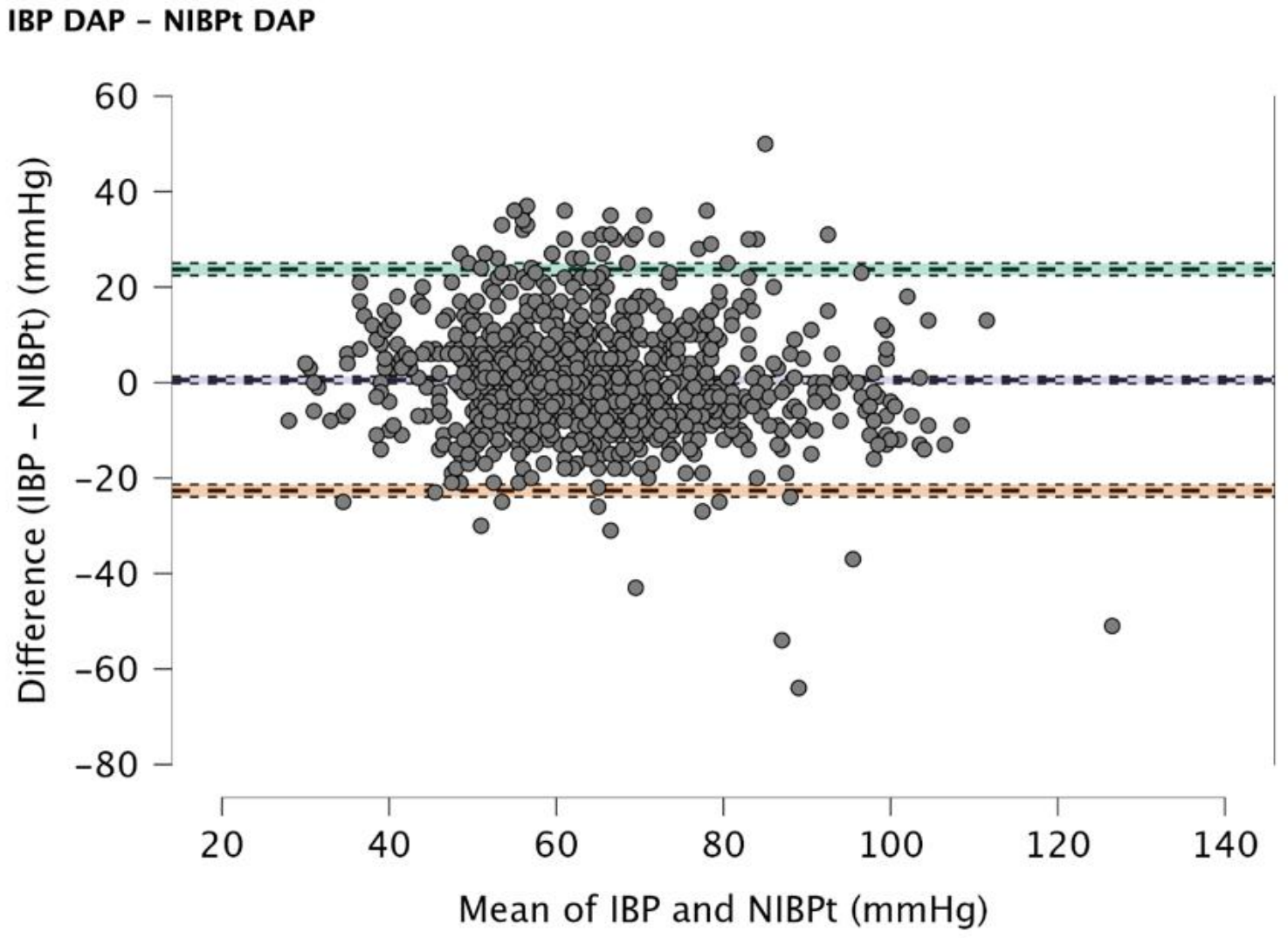

Bland–Altman Tables

IBP SAP – NIBPt SAP

Bias & Limits	Point Value	Lower 95% CI	Upper 95% CI
Mean difference + 1.96 SD	49.21	47.083	51.33
Mean difference	10.66	9.437	11.89
Mean difference – 1.96 SD	−27.88	−30.005	−25.76

IBP MAP – NIBPt MAP

Bias & Limits	Point Value	Lower 95% CI	Upper 95% CI
Mean difference + 1.96 SD	24.937	23.817	26.056
Mean difference	4.628	3.982	5.275
Mean difference – 1.96 SD	−15.680	−16.800	−14.561

IBP DAP – NIBPt DAP

Bias & Limits	Point Value	Lower 95% CI	Upper 95% CI
Mean difference + 1.96 SD	23.714	22.437	24.992
Mean difference	0.535	−0.202	1.273
Mean difference – 1.96 SD	−22.644	−23.921	−21.366

Supplementary Figure 1B: ***Bland–Altman plots comparing invasive arterial blood pressure (IBP) with tongue-based oscillometric blood pressure (NIBPt) in dogs >10 kg for systolic arterial blood pressure (SAP), mean arterial blood pressure (MAP) and diastolic arterial blood pressure (DAP).*** *Points represent paired measurements. The central dashed line is the mean difference (bias = IBP − NIBPt); outer dashed lines are the mean difference ±1.96 standard deviations (SD) of the differences (95% limits of agreement, LoA). Thin flanking dashes indicate the 95% confidence intervals (CIs) for the bias and each LoA. Axes in mmHg. The Bland–Altman summary table lists the point estimate and 95% CI for the mean difference and for each LoA (presented as "mean difference ±1.96 SD").*

Bland-Altman Plots for Non-chondrodystrophic Breeds (N = 53)

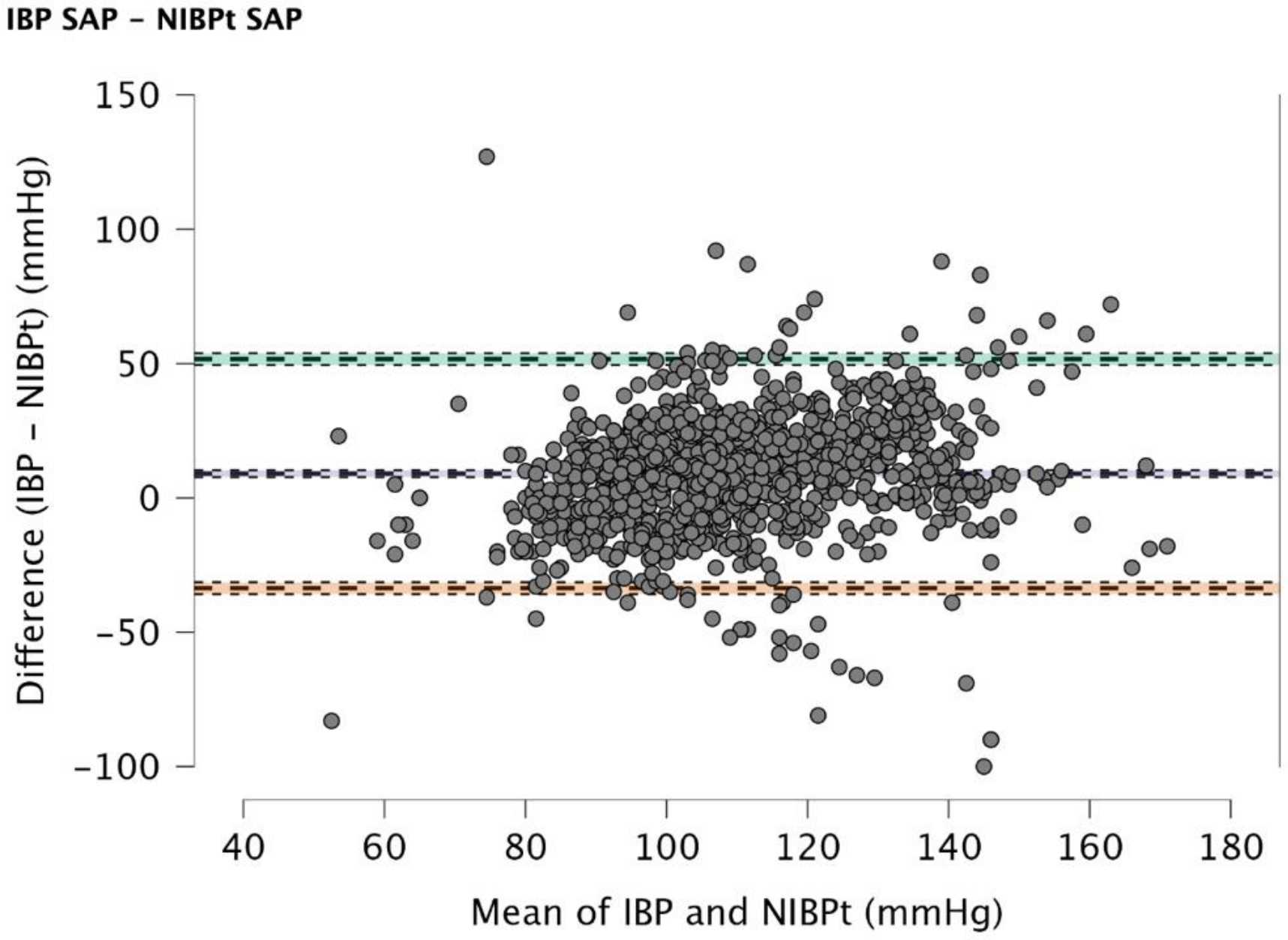

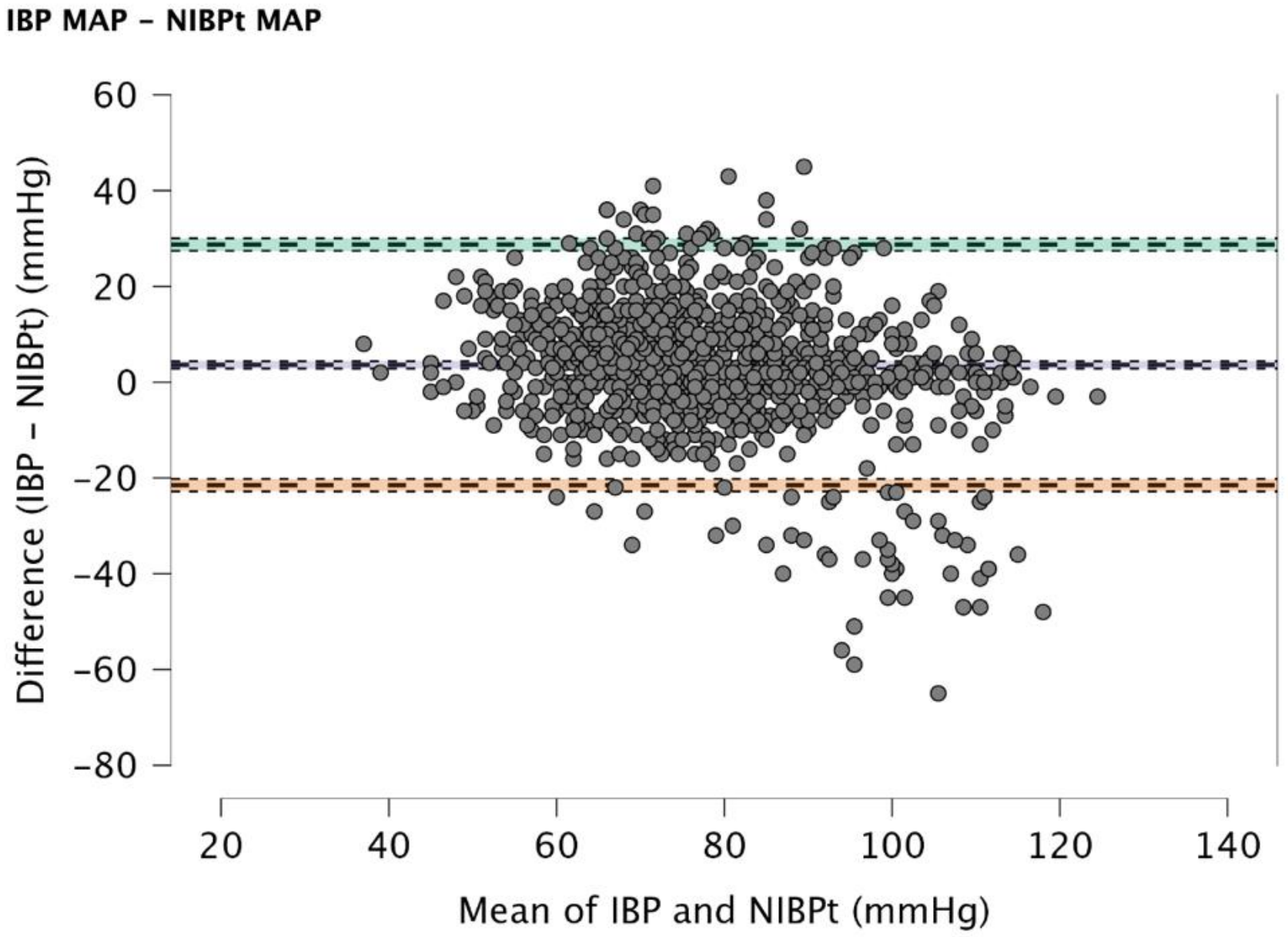

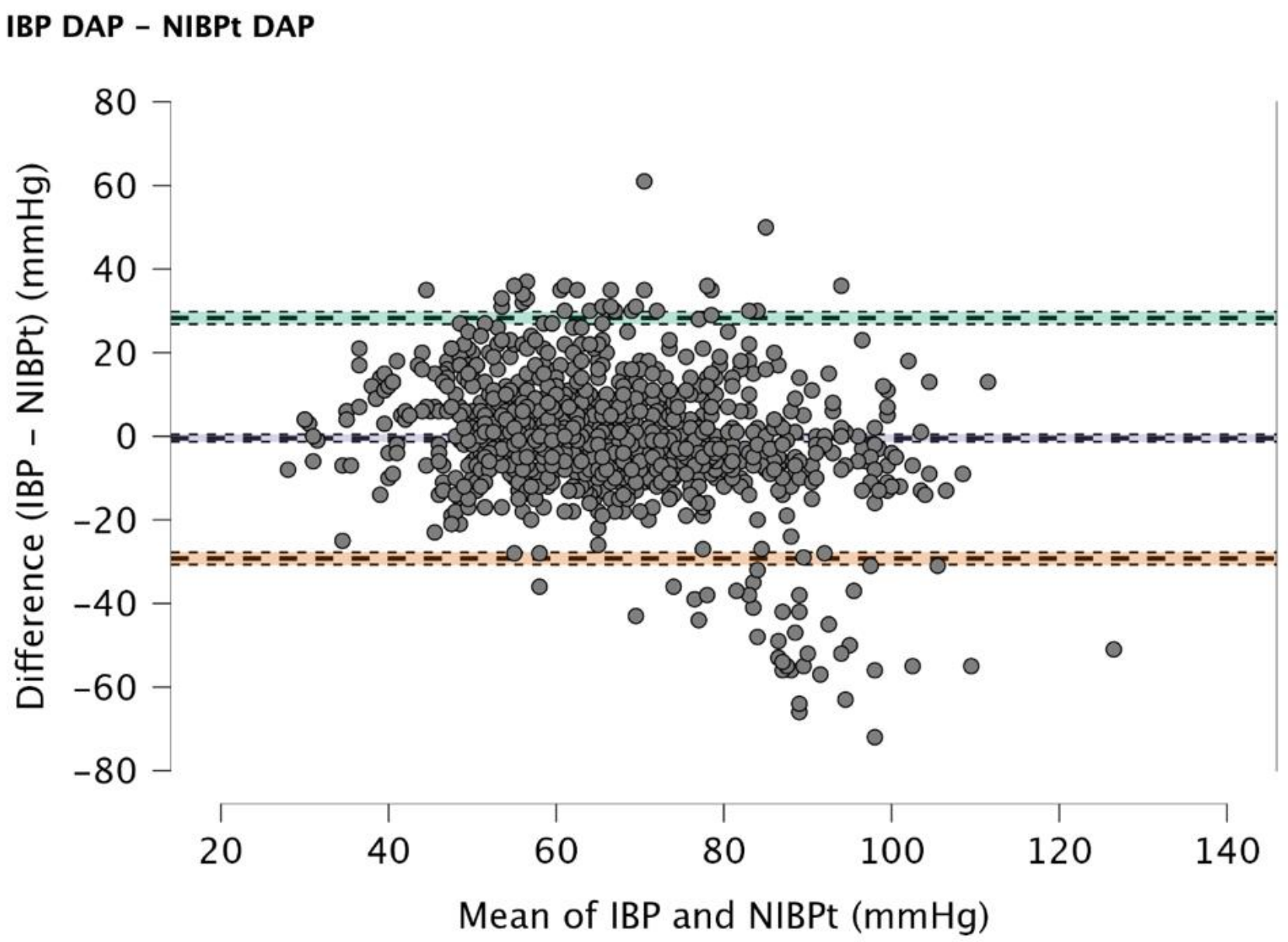

Bland–Altman Tables

IBP SAP – NIBPt SAP

Bias & Limits	Point Value	Lower 95% CI	Upper 95% CI
Mean difference + 1.96 SD	51.662	49.465	53.86
Mean difference	9.022	7.753	10.29
Mean difference − 1.96 SD	−33.618	−35.816	−31.42

IBP MAP – NIBPt MAP

Bias & Limits	Point Value	Lower 95% CI	Upper 95% CI
Mean difference + 1.96 SD	28.758	27.463	30.053
Mean difference	3.628	2.880	4.376
Mean difference − 1.96 SD	−21.502	−22.797	−20.207

IBP DAP – NIBPt DAP

Bias & Limits	Point Value	Lower 95% CI	Upper 95% CI
Mean difference + 1.96 SD	28.336	26.852	29.820
Mean difference	−0.454	−1.311	0.403
Mean difference − 1.96 SD	−29.244	−30.728	−27.761

Supplementary Figure 2A: ***Bland–Altman plots comparing invasive arterial blood pressure (IBP) with tongue-based oscillometric blood pressure (NIBPt) in non-chondrodystrophic dogs for systolic arterial blood pressure (SAP), mean arterial blood pressure (MAP) and diastolic arterial blood pressure (DAP).*** *Points represent paired measurements. The central dashed line is the mean difference (bias = IBP − NIBPt); outer dashed lines are the mean difference ±1.96 standard deviations (SD) of the differences (95% limits of agreement, LoA). Thin flanking dashes indicate the 95% confidence intervals (CIs) for the bias and each LoA. Axes in mmHg. The Bland–Altman summary table lists the point estimate and 95% CI for the mean difference and for each LoA (presented as "mean difference ±1.96 SD").*

Bland-Altman Plots for Chondrodystrophic Breeds (N = 36)

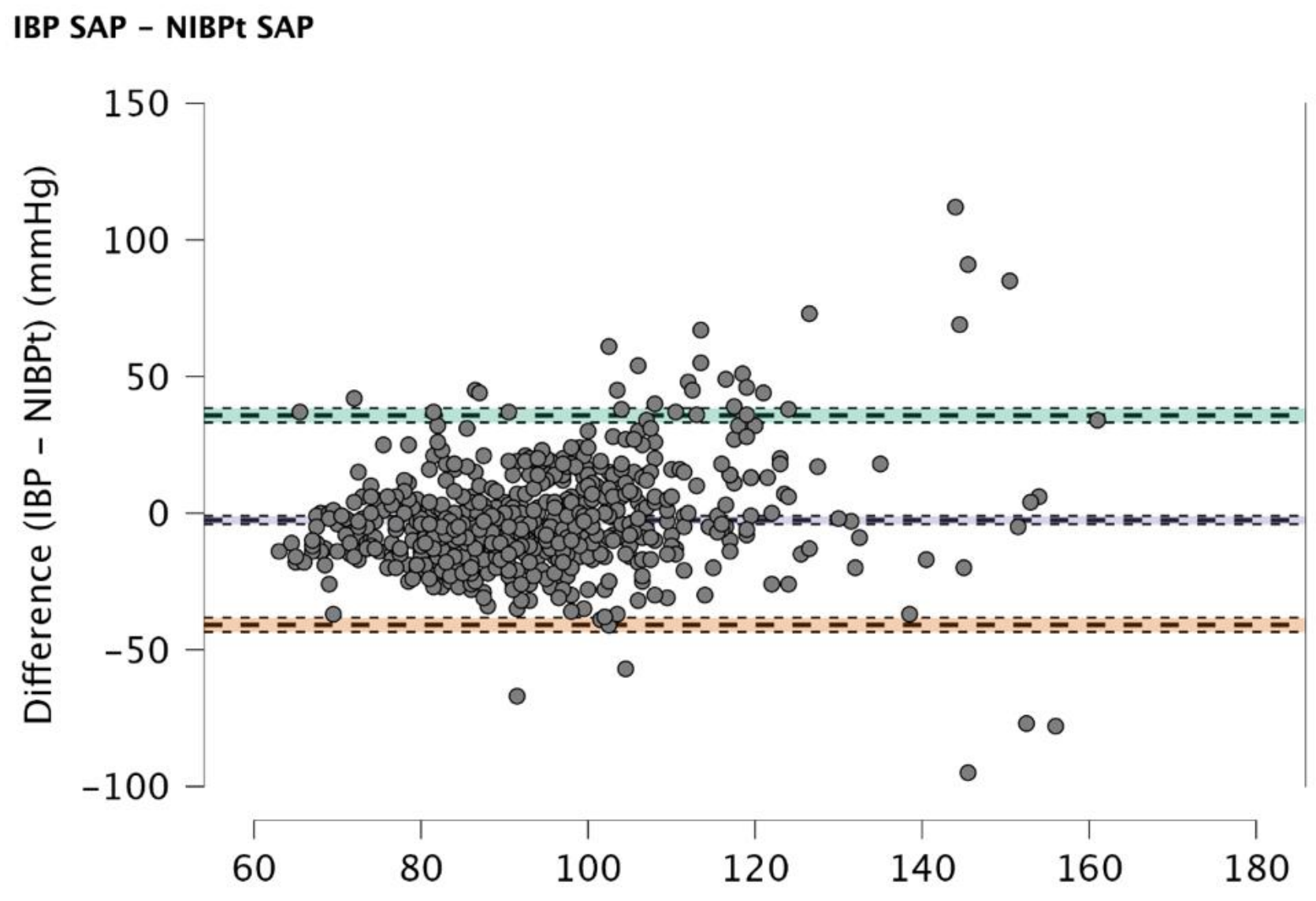

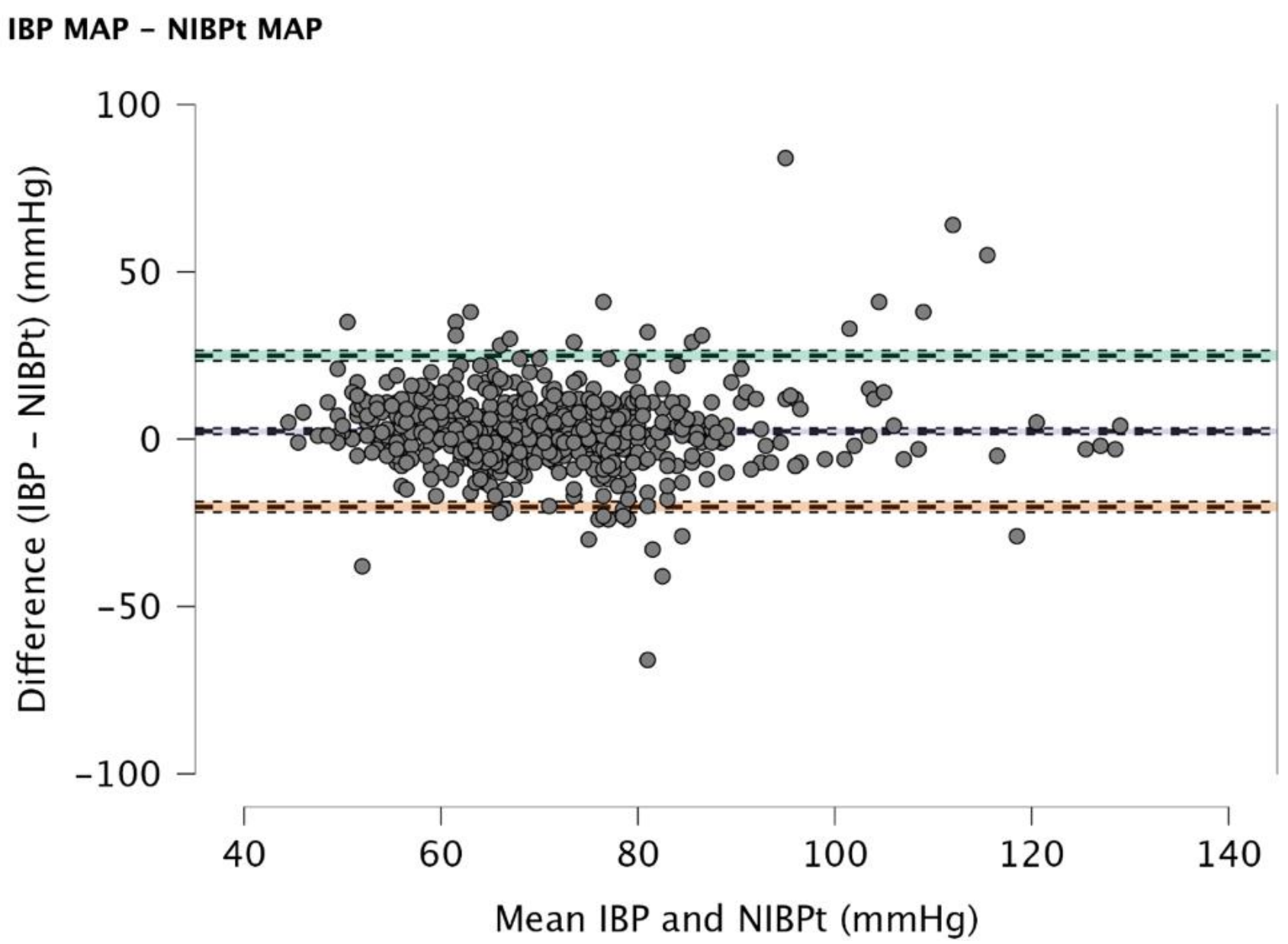

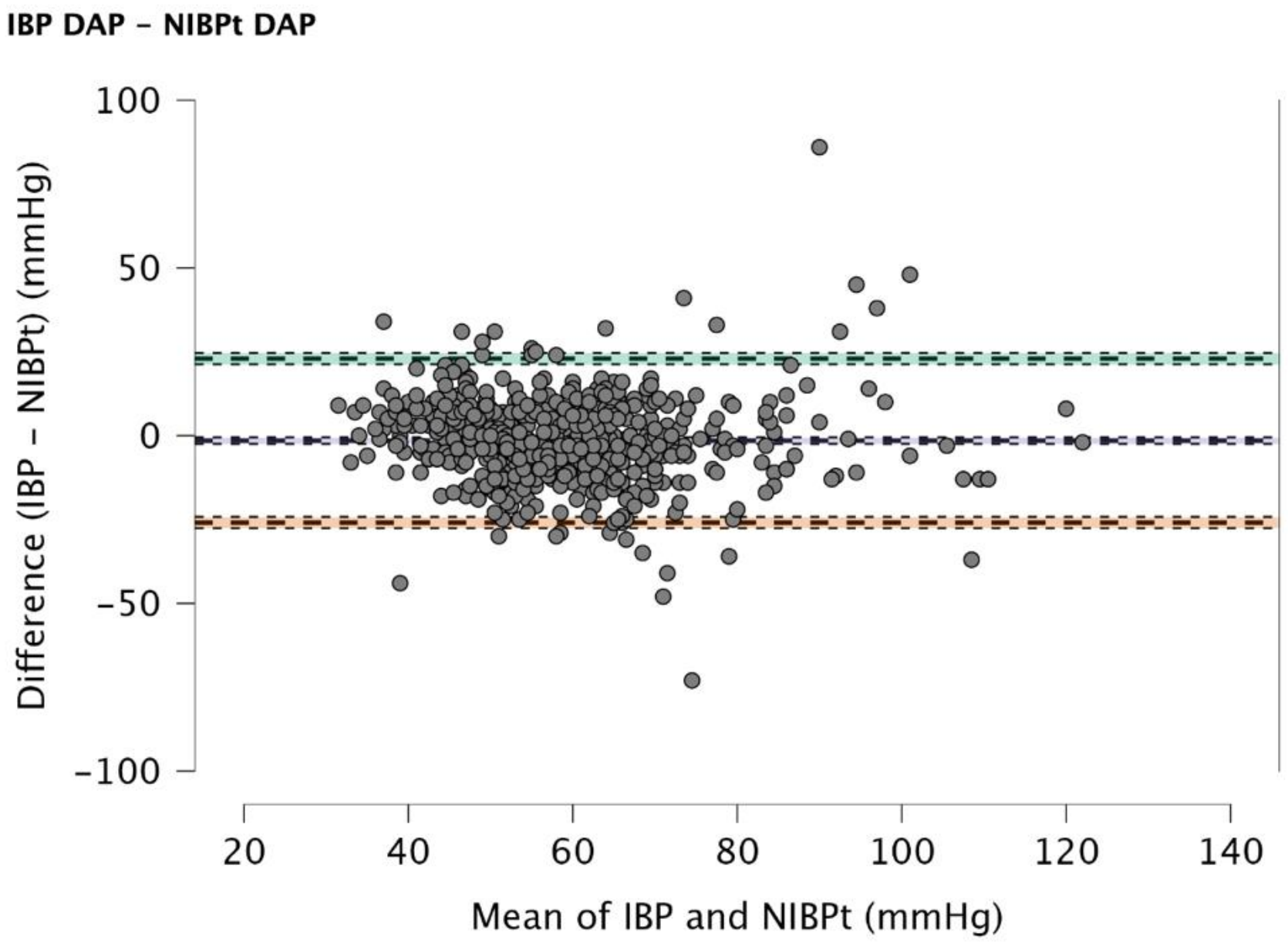

Bland–Altman Tables

IBP SAP – NIBPt SAP

Bias & Limits	Point Value	Lower 95% CI	Upper 95% CI
Mean difference + 1.96 SD	35.778	33.166	38.391
Mean difference	−2.551	−4.059	−1.042
Mean difference – 1.96 SD	−40.880	−43.493	−38.268

IBP MAP – NIBPt MAP

Bias & Limits	Point Value	Lower 95% CI	Upper 95% CI
Mean difference + 1.96 SD	24.953	23.413	26.493
Mean difference	2.356	1.467	3.246
Mean difference – 1.96 SD	−20.240	−21.780	−18.700

IBP DAP – NIBPt DAP

Bias & Limits	Point Value	Lower 95% CI	Upper 95% CI
Mean difference + 1.96 SD	22.943	21.276	24.610
Mean difference	−1.490	−2.452	−0.528
Mean difference – 1.96 SD	−25.923	−27.590	−24.256

Supplementary Figure 2B: ***Bland–Altman plots comparing invasive arterial blood pressure (IBP) with tongue-based oscillometric blood pressure (NIBPt) in chondrodystrophic dogs for systolic arterial blood pressure (SAP), mean arterial blood pressure (MAP) and diastolic arterial blood pressure (DAP).*** *Points represent paired measurements. The central dashed line is the mean difference (bias = IBP − NIBPt); outer dashed lines are the mean difference ±1.96 standard deviations (SD) of the differences (95% limits of agreement, LoA). Thin flanking dashes indicate the 95% confidence intervals (CIs) for the bias and each LoA. Axes in mmHg. The Bland–Altman summary table lists the point estimate and 95% CI for the mean difference and for each LoA (presented as "mean difference ±1.96 SD").*
