# Supplementary material for: Comparison of lingual oscillometric and invasive blood pressure measurement in dogs under general anaesthesia
Source: J Small Anim Pract. 2026 Jul 12;67(11):1082–90. doi: 10.1111/jsap.70163 (PMC13728962; doi:10.1111/jsap.70163)
Supplement: Supplementary file 2 [file JSAP-67-1082-s001.docx]

## **Supplementary Material:** Statistical analysis and diagnostics (mixed-effects proportional bias).

**Overview:**

This supplementary material provides full model specifications, interaction tests, and diagnostic summaries for the mixed-effects proportional-bias analyses described in the manuscript. It complements the Bland–Altman summaries in the main text.

Model 1: Proportional bias without effect modification

SAP:

For every 1 mmHg increase in mean SAP, IBP overestimates NIBPt by ~0.22 mmHg on average. There is strong statistical evidence for this since 95% CI is relatively narrow. No strong statistical evidence that there is proportional bias for DAP and MAP (95% CI crosses 0).

| **Outcome** | **Estimate (95%CI)** |
| --- | --- |
| SAP | 0.22 (0.07, 0.37) |
| MAP | -0.09 (-0.21, 0.02) |
| DAP | -0.11 (-0.24, 0.02) |

Model 2: Proportional bias with effect modification by body weight

There is very strong statistical evidence that proportional bias differed by body weight for the SAP outcome when testing for effect modification using likelihood ratio test. Specifically, IBP overestimates NIBPt by 0.44 per 1 mmHg increase in mean SAP in dogs larger than 10kg. Meanwhile, no proportional bias was observed in dogs less than 10kg. Proportional bias did not differ by body weight in MAP and DAP outcomes.

| **Outcome** | **Weight group** | **Estimate (95% CI)** | **P-values for interaction (LRT)** |
| --- | --- | --- | --- |
| SAP | >10 kg | 0.44 (0.24, 0.65) | 0.001 |
| SAP | ≤10 kg | -0.02 (-0.22, 0.18) |  |
| MAP | >10 kg | -0.06 (-0.23, 0.11) | 0.44 |
| MAP | ≤10 kg | -0.13 (-0.29, 0.04) |  |
| DAP | >10 kg | -0.07 (-0.25, 0.11) | 0.43 |
| DAP | ≤10 kg | -0.15 (-0.33, 0.02) |  |

Model 3: Proportional bias with effect modification by phenotype (chondrodystrophic/ non-chondrodystrophic breed)

There is strong statistical evidence showing proportional bias differed by phenotype for the SAP outcome. IBP overestimates NIBPt by 0.27 per 1 mmHg increase in mean SAP in non-chondrodystrophic breed. Proportional bias did not differ by breed for the DAP and MAP outcomes when testing for effect modification using likelihood ratio test.

| **Outcome** | **Phenotype** | **Estimate (95% CI)** | **P-values for interaction (LRT)** |
| --- | --- | --- | --- |
| SAP | Chondrodystrophic | 0.12 (-0.12, 0.36) | 0.01 |
| SAP | Non-chondrodystrophic | 0.27 (0.07, 0.46) |  |
| MAP | Chondrodystrophic | 0.0005 (-0.18, 0.18) | 0.10 |
| MAP | Non-chondrodystrophic | -0.16 (-0.31, -0.01) |  |
| DAP | Chondrodystrophic | -0.01 (-0.21, 0.19) | 0.17 |
| DAP | Non-chondrodystrophic | -0.18 (-0.35, -0.02) |  |
