# Supplementary material for: Comparison of lingual oscillometric and invasive blood pressure measurement in dogs under general anaesthesia
Source: J Small Anim Pract. 2026 Jul 12;67(11):1082–90. doi: 10.1111/jsap.70163 (PMC13728962; doi:10.1111/jsap.70163)
Supplement: Supplementary file 3 [file JSAP-67-1082-s003.docx]

| Stratum | Dogs (N) | Measure | Bias (mmHg) | Precision (mean bias SD) | LoA low | LoA high | % of dogs ≤10 mmHg* | % of dogs ≤20 mmHg* | Meets ACVIM† | Meets AAMI/ESH/ISO‡ |
| --- | --- | --- | --- | --- | --- | --- | --- | --- | --- | --- |
| All dogs | 89 | SAP | 5.1 | 17.6 | -29.4 | 39.5 | 22.5 | 73 | No | No |
|  |  | MAP | 2.8 | 8.4 | -13.8 | 19.3 | 73 | 93.3 | **Yes** | No |
|  |  | DAP | -1.4 | 8.5 | -18.1 | 15.3 | 67.4 | 93.3 | **Yes** | No |
| Weight ≤10 kg | 45 | SAP | -1.9 | 17.1 | -35.4 | 31.7 | 24.4 | 82.2 | No | No |
|  |  | MAP | 1.1 | 9.9 | -18.2 | 20.5 | 71.1 | 88.9 | **Yes** | No |
|  |  | DAP | -2.9 | 10.1 | -22.7 | 16.9 | 64.4 | 88.9 | **Yes** | No |
| Weight >10 kg | 44 | SAP | 12.1 | 15.3 | -17.8 | 42.1 | 20.5 | 63.6 | No | No |
|  |  | MAP | 4.5 | 6.4 | -8 | 17 | 75 | 97.7 | **Yes** | No |
|  |  | DAP | 0.1 | 6.4 | -12.4 | 12.6 | 70.5 | 97.7 | **Yes** | No |
| Chondrodystrophic | 36 | SAP | -2.3 | 14.3 | -30.4 | 25.7 | 27.8 | 86.1 | No | No |
|  |  | MAP | 2.1 | 7.6 | -12.8 | 17 | 77.8 | 94.4 | **Yes** | No |
|  |  | DAP | -1.7 | 7.4 | -16.3 | 12.8 | 66.7 | 94.4 | **Yes** | No |
| Not Chondrodystrophic | 53 | SAP | 10.1 | 18 | -25.1 | 45.3 | 18.9 | 64.2 | No | No |
|  |  | MAP | 3.3 | 9 | -14.4 | 20.9 | 69.8 | 92.5 | **Yes** | No |
|  |  | DAP | -1.2 | 9.3 | -19.4 | 17 | 67.9 | 92.5 | **Yes** | No |

Supplementary Table S1: **Subject-level (per-dog) Bland–Altman agreement between invasive blood pressure (IBP) and tongue-based oscillometric blood pressure (NIBPt):** Bias is defined as IBP − NIBPt (mmHg). For each dog and pressure (SAP, MAP, DAP), the per-dog mean difference was computed; bias, precision (SD), and 95% limits of agreement (LoA) were derived from these per-dog means. “% of dogs ≤10/≤20 mmHg” denotes the proportion of dogs whose **per-dog mean absolute difference** from IBP was within the stated threshold. ACVIM and AAMI/ESH/ISO columns are provided for context at the subject level; formal device-level validation was performed on the pair-level dataset (Table 3). Strata include all dogs and subgroups by body size (≤10 kg vs >10 kg) and phenotype (chondrodystrophic vs non-chondrodystrophic).
**Footnotes.**
*Percentages computed from per-dog mean absolute differences (IBP − NIBPt).
†“Meets ACVIM” defined as ≥50% of dogs ≤10 mmHg **and** ≥80% of dogs ≤20 mmHg; shown here as a descriptive, cluster-level complement to pair-level validation.
‡“Meets AAMI/ESH/ISO” shown descriptively; formal AAMI/ESH/ISO device accuracy was assessed on pair-level data and not met in any stratum
